# Unpopularity of Private Nursing with Hospital Staffs

**Published:** 1919-11-08

**Authors:** 


### UNPOPULARITY OF PRIVATE NURSING WITH HOSPITAL STAFFS.

By F. A. G.

ONE often wonders why private nursing is such an unpopular branch of the nursing profession with the present-day nurse! I have come into contact with many nurses of late, who, upon being asked the question as they neared the completion of their training, "Which branch of the profession do you propose to take up?" reply, "I should like to stay in hospital!"

Now, whilst reforms are being discussed and reconstruction is taking place in our profession (chiefly in the hospital world) the moment seems opportune for the discussion of conditions of private nursing and to suggest schemes whereby the life may be made more attractive to a nurse. I intend to confine my remarks entirely to the hospital private nursing staff. Until recently only the ambitious few had any real desire to stay in hospital, but now that conditions are being so much improved with regard to salaries, off-duty time, and comforts, the nurse realises that she is better off in hospital, for few of these improvements are finding their way into other branches of the profession as yet. These material advantages will, and must, be taken into consideration by the newly trained nurse, who is about to take her place in the competition of the nursing world, however great a love she may have for her vocation.

Many newly trained nurses find some difficulty in deciding which branch of the profession will give them the greatest scope for their own particular talents, and it is in this direction that the hospital private nursing staff could make itself attractive if improvements were made. One of the chief reasons why private nursing is so unpopular amongst the hospital staff is that the private nurse, as a rule, has no little corner which she may call her own except perhaps a locker, wardrobe, or drawer, wherein are crammed all her worldly possessions; sometimes she has not even this, but "lives in her trunk," whilst the ward sisters, who were perhaps her colleagues in training or may be even her juniors, have their own bedrooms, usually furnished very comfortably, occasionally as bed-sitting-rooms, in which they are able to create a homely and personal atmosphere. The probationers too have their own bedrooms (for a few months at a time, at any rate), and when their periods of night-duty are finished they usually retain one room until the completion of training, and they also are able to introduce personal and homely touches, and regard their rooms as their own special property.

The private nurse has not this comfort to look forward to when coming in from a case, but is given a bed wherever there happens to be an empty room. Sometimes certain rooms on a floor are reserved for the private staff, but she cannot keep her own; nor can she unpack more of her possessions than are absolutely necessary, because she must be ready for another case at short notice. Consequently her room is never home to her.

Another reason against private nursing is that the nurse is so often the forgotten member of the staff until she is due in from a case. When she comes in, too often all her personal friends are away at cases. The result is that she feels like a "ship out of water"; generally unwanted and in the way. She is thoroughly miserable and longs with all her heart for the next case to come along. She is a trained nurse, and therefore should be treated as the sister's equal, and given the same advantages and comfort so far as is possible. Her responsibilities may not be quite so great, but in her own sphere she is doing equally useful work for the hospital.

If it were possible, one would like to see a private nursing staff run in conjunction with every training-school. It would not only afford the nurses an opportunity of enlarging their experience under the protection of their training-school, but it would be also a lucrative branch of work. It would serve as an advertisement to the hospital, and often attract subscriptions and legacies, especially if only the right kind of nurse were retained on the staff. This is a most important point, for it must not be forgotten that the public very often base their estimate of a hospital on the kind of nurses it sends out, and *one* nurse may make or mar the reputation of a hospital among a large community. Nurses should be taught to realise their responsibility in this respect; to be loyal at all times to their hospital and profession, and careful to do nothing which will bring it into disrepute.

This branch of work, however, if it is to be attractive, must not be run solely as a source of income. The nurse's interests should be the first consideration after the patients, and only when everything possible has been done for them should any part of the income be diverted to hospital use.

